# C_4_ anatomy can evolve via a single developmental change

**DOI:** 10.1111/ele.13191

**Published:** 2018-12-17

**Authors:** Marjorie R. Lundgren, Luke T. Dunning, Jill K. Olofsson, Jose J. Moreno‐Villena, Jacques W. Bouvier, Tammy L. Sage, Roxana Khoshravesh, Stefanie Sultmanis, Matt Stata, Brad S. Ripley, Maria S. Vorontsova, Guillaume Besnard, Claire Adams, Nicholas Cuff, Anthony Mapaura, Matheus E. Bianconi, Christine M. Long, Pascal‐Antoine Christin, Colin P. Osborne

**Affiliations:** ^1^ Department of Animal and Plant Sciences University of Sheffield Western Bank Sheffield S10 2TN UK; ^2^ Department of Ecology and Evolutionary Biology University of Toronto 25 Willcocks Street Toronto ON M5S 3B2 Canada; ^3^ Botany Department Rhodes University Grahamstown 6139 South Africa; ^4^ Comparative Plant and Fungal Biology Royal Botanic Gardens Kew Richmond Surrey TW9 3AB UK; ^5^ Laboratoire Évolution & Diversité Biologique (EDB UMR5174) Université de Toulouse CNRS ENSFEA UPS IRD 118 route de Narbonne 31062 Toulouse France; ^6^ Northern Territory Herbarium Department of Environment and Natural Resources PO Box 496 Palmerston NT 0831 Australia; ^7^ National Herbarium and Botanic Garden Harare Zimbabwe; ^8^ Department of Primary Industry and Fisheries Northern Territory Government Darwin NT 0801 Australia; ^9^ Present address: Lancaster Environment Centre Lancaster University Lancaster LA1 4YQ UK

**Keywords:** *Alloteropsis*, bundle sheath, C_3_‐C_4_ intermediate, C_4_ photosynthesis, evolution, grass, leaf anatomy, mesophyll, vein density

## Abstract

C_4_ photosynthesis is a complex trait that boosts productivity in warm environments. Paradoxically, it evolved independently in numerous plant lineages, despite requiring specialised leaf anatomy. The anatomical modifications underlying C_4_ evolution have previously been evaluated through interspecific comparisons, which capture numerous changes besides those needed for C_4_ functionality. Here, we quantify the anatomical changes accompanying the transition between non‐C_4_ and C_4_ phenotypes by sampling widely across the continuum of leaf anatomical traits in the grass *Alloteropsis semialata*. Within this species, the only trait that is shared among and specific to C_4_ individuals is an increase in vein density, driven specifically by minor vein development that yields multiple secondary effects facilitating C_4_ function. For species with the necessary anatomical preconditions, developmental proliferation of veins can therefore be sufficient to produce a functional C_4_ leaf anatomy, creating an evolutionary entry point to complex C_4_ syndromes that can become more specialised.

## Introduction

The vast majority of plants use C_3_ photosynthesis, but some lineages evolved the C_4_ pathway to overcome environmentally induced limitations on carbon fixation (Ehleringer *et al*. 1991; Sage *et al*. 2011). Net carbon fixation by C_3_ photosynthesis is decreased in warm, high light, arid and saline environments that lower CO_2_ concentrations within the leaf and increase photorespiration, the process initiated when O_2_ instead of CO_2_ is fixed by the enzyme Rubisco (Chollet & Ogren 1975). To circumvent the losses of carbon and energy caused by photorespiration, the C_4_ pathway spatially separates the initial fixation of carbon and its assimilation by Rubisco across two leaf compartments, thereby concentrating CO_2_ at the enzyme's active site to promote CO_2_ rather than O_2_ fixation (Downton & Tregunna 1968; Hatch 1976). A number of anatomical and biochemical functions must work in concert to sustain the high fluxes of the C_4_ cycle, and comparisons of average C_4_ and C_3_ plants suggest that the evolution of the C_4_ phenotype required a large number and scale of changes (Hattersley 1984). Despite this apparent complexity, the C_4_ trait evolved many times independently (Sage *et al*. 2011). Resolving this paradox requires the quantitative distinction of changes that were involved in the evolutionary transition from C_3_ to C_4_, from those that preceded or followed it.

**Figure 1:**
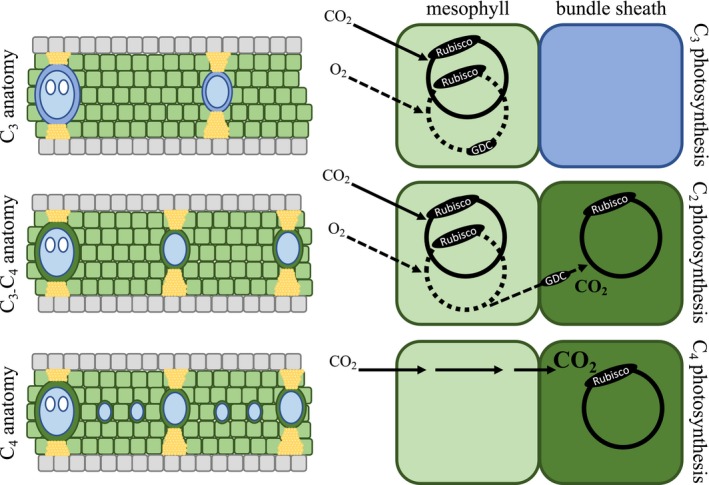
Schematic of leaf anatomy and photosynthetic pathway in C_3_, C_3_‐C_4_ and C_4_ grasses. In C_3_ plants, CO_2_ assimilation via the Calvin–Benson cycle (solid black circle) and CO_2_ release via photorespiration (dashed black circle) both occur in mesophyll cells (light green). C_3_ leaves consequently have larger areas of mesophyll tissue than bundle sheath tissue, where no photosynthetic activity occurs. C_3_‐C_4_ plants use an intermediate physiology called C_2_ photosynthesis, where the Calvin‐Benson cycle occurs in mesophyll cells, like in C_3_ plants. However, because glycine decarboxylase (GDC) is specifically localised to bundle sheath cells in these plants, the photorespiratory cycle is split across these two cell types, creating a weak CO_2_‐concentrating mechanism, where CO_2_ is released in the bundle sheath and can be reassimilated via the Calvin cycle. C_2_ photosynthesis, therefore, requires large areas of mesophyll for photosynthesis via an initial Calvin‐Benson cycle, but also close contact between mesophyll and bundle sheath cells for the photorespiratory CO_2_ pump. C_4_ plants have a strong CO_2_ concentrating mechanism whereby CO_2_ is biochemically shuttled from the mesophyll into the bundle sheath. The high CO_2_ concentration in the bundle sheath largely avoids oxygenation and thus, photorespiration. Photosynthesis via the C_4_ cycle therefore requires large areas of bundle sheath tissue, but less mesophyll, which can be achieved via the insertion of minor veins. Dark blue, bundle sheath lacking chloroplasts; dark green, bundle sheaths with chloroplasts; light green, mesophyll cells; yellow, extraxylary fibres/bundle sheath extensions; grey, epidermal cells; light blue, veins; white, metaxylem.

In most C_4_ plants, carbon fixation within leaf mesophyll tissue (M) is used to concentrate CO_2_ and boost Rubisco activity within bundle sheath tissue (BS), whereas Rubisco in C_3_ plants operates within the M where it depends on atmospheric CO_2_ diffusion (Fig. 1; Brown 1975; Hattersley *et al*. 1977; Hatch 1987). Efficient C_4_ leaves require large BS volumes to accommodate the necessary photosynthetic organelles, including chloroplasts containing abundant Rubisco, and a small distance between M and BS compartments to allow the rapid transfer of metabolites (Fig. 1; Hattersley & Watson 1975; Lundgren *et al*. 2014). These traits vary among C_3_ plant lineages, and in grasses, C_4_ photosynthesis evolved only within those groups with large fractions of BS (Christin *et al*. 2013; Lundgren *et al*. 2014). Comparisons of multiple C_4_ lineages with their C_3_ relatives indicate that the evolution of C_4_ leaf anatomy involved ultrastructural rearrangements and further decreases to the relative volume of M compared to BS tissue (Hattersley 1984; Dengler *et al*. 1994; McKown & Dengler 2007; Christin *et al*. 2013). These properties can be achieved via a variety of leaf structural modifications, allowing C_4_ anatomy to be realised differently each time it evolved, in some cases involving the use of different tissue types for the C_4_ BS function (Brown 1975; Soros & Dengler 2001; Christin *et al*. 2013; Freitag & Kadereit 2014; Lundgren *et al*. 2014). While the differences between a diverse range of C_3_ and C_4_ species are well known, the minimum set of leaf anatomical modifications required to carry out C_4_ photosynthesis remains to be established.

The grass *Alloteropsis semialata* (R.Br.) Hitchc. provides an outstanding system to capture the early events during C_4_ evolution because it includes genetically divergent C_4_ individuals as well as a diversity of non‐C_4_ plants encompassing C_3_ and C_3_‐C_4_ intermediate phenotypes (Ellis 1974; Lundgren *et al*. 2016), which emerged in the palaeotropics (Lundgren *et al*. 2015). The inner sheath (i.e., the mestome sheath), which is present in all C_3_ grasses, has been co‐opted for the C_4_ BS function in *A. semialata*. Previous studies have compared leaf properties among C_4_ and non‐C_4_ leaves of a few *A. semialata* accessions (Ellis 1974; Frean *et al*. 1983; Ueno & Sentoku 2006; Lundgren *et al*. 2016; Dunning *et al*. 2017), but a broader sampling is required to establish which properties are unique to each photosynthetic type.

The primary focus of this study is to compare leaf anatomy in accessions spanning the diversity of each photosynthetic type to distinguish the structural diversifications that occurred before, during and after C_4_ emergence in this species. We hypothesise that the properties that predate C_4_ evolution will be shared by at least some of the non‐C_4_ individuals, while those that happened after C_4_ evolution in a phase of subsequent adaptation will be restricted to a subset of the C_4_ populations. Properties unique to, and common among all, C_4_ accessions represent those that were involved in the initial transition to a C_4_ physiology. We conducted a large scan of the diversity within the species using traits linked to the number and size of different cell types and used controlled growth experiments to verify that anatomical differences are not environmentally induced. This evaluation of the gross leaf morphology was accompanied by a focused study in some individuals to identify ultrastructural changes that may also differ between C_4_ and non‐C_4_ accessions. Overall, our work shows that a complex trait of large ecological significance can evolve via a few key developmental changes.

## Materials and methods

### Characterising photosynthetic types

Photosynthetic type was determined by a combination of stable isotope and CO_2_ compensation point (CCP) data (Table S1; Data set S1), as previously described (Lundgren *et al*. 2016). The carbon isotope composition of plant tissues (δ^13^C) distinguishes photosynthetic types (von Caemmerer *et al*. 2014), such that plants with δ^13^C values higher than −17‰ were considered to have a fully functioning C_4_ system, while those with values lower than this threshold were considered either C_3_ or C_3_‐C_4_. CCPs were used to distinguish C_3_‐C_4_ from C_3_ plants and to support the δ^13^C results. The CCP indicates the CO_2_ concentration within the leaf at which CO_2_ assimilation via photosynthesis equals CO_2_ loss via photorespiration and respiration. As less CO_2_ is ultimately lost to photorespiration in C_3_‐C_4_ plants, they have very low CCPs compared to C_3_ plants. Thus, non‐C_4_ plants with CCPs greater than or equal to 35 μmol mol^−1^ were classified as C_3_, while those < 35 μmol mol^−1^ were classified as C_3_‐C_4_. CCPs were calculated on 27 living accessions (6 C_3_, 4 C_3_‐C_4_ and 17 C_4_), following published protocols (Bellasio *et al*. 2016a,b; Lundgren *et al*. 2016). Non‐C_4_ accessions for which live material was unavailable were assumed to have the same photosynthetic type as their closest relatives, as identified by phylogenetic relationships (Table S1).

### Leaf samples

Fifty *Alloteropsis semialata* (R.Br.) Hitchc. accessions distributed across the species’ geographic range, including 17 C_3_, 6 C_3_‐C_4_ and 27 C_4_, were used to assess intraspecific anatomical variation. Leaf samples from 44 of the 50 accessions were collected from their original field site and preserved until embedding was possible. For the remaining six accessions, leaf samples were taken from plants grown under controlled environment conditions as in Lundgren *et al*. (2016). For all samples, leaf pieces 3–5 mm in length were embedded in methacrylate embedding resin (Technovit 7100, Heraeus Kulzer GmbH, Wehrhein, Germany), sectioned 6–8 μm thick on a manual rotary microtome (Leica Biosystems, Newcastle, UK) and stained with Toluidine Blue O (Sigma‐Aldrich, St. Louis, MO, USA). Stained leaf sections were imaged using microscopy imaging software with a camera mounted on a microscope (Cell A, Olympus DP71 and Olympus BX51, respectively; Olympus, Hamburg, Germany) and the images were stitched together using DoubleTake (v2.2.9, Echo One, Frederikssund, Denmark).

### Leaf anatomy measurements

Anatomical traits were measured using ImageJ (Fig. S1; Schneider *et al*. 2012) from the cross section of a single leaf segment from the centre of the leaf blade, avoiding segments immediately adjacent to the midrib and lateral edges of the cross section. Vein orders were distinguished following Renvoize (1987). A single segment was defined as the leaf area falling between two secondary veins, which are large veins with metaxylem. Tertiary and minor veins (e.g. quaternary and quinary orders) lack metaxylem. In this species, the extraxylary fibres that flank both the adaxial and abaxial edges of tertiary veins distinguish them from higher order minor veins, which can be flanked by fibres on one side only (Fig. S1).

The cross‐sectional area of the whole segment, combining M, BS, epidermis and bulliform cells, extraxylary fibres and BS extensions as well as any transverse veins or tear spaces was measured. For all accessions, the total BS (i.e. the inner sheath; the compartment used for the Calvin cycle in C_4_ *A. semialata*), outer sheath and vein areas were measured separately for secondary, tertiary and any minor veins. The area of M tissue was calculated as the total area remaining after accounting for all other tissue types. In addition, the cross‐sectional area of individual M and BS cells (hereafter ‘size’) was measured (Fig. S1). Although the depth of individual cells can vary, it is their cross‐sectional areas, and not their three‐dimensional volumes, that primarily influence the proportion of each tissue in the leaf.

### Linear discriminant analysis

We used a linear discriminant analysis (LDA) to explain the variation between photosynthetic types (i.e. the test maximises between‐group variance while minimising within‐group variance). We performed the LDA on the 50 accessions with leave‐one‐out cross‐validation and then bootstrapping over 100 runs, using the MASS package in R (Venables & Ripley 2002). Prior probabilities were based on the relative sample size of the categorical variable (i.e. 0.34, 0.12 and 0.54 for C_3_, C_3_‐C_4_ and C_4_ groups respectively). We chose predictor variables that were likely to influence the M : BS ratio, including the number of M cells between major veins, average size of individual M cells, number of minor veins per segment, leaf thickness and the average size of BS cells on tertiary veins.

To test the generality of our findings from *A. semialata*, we carried out an equivalent LDA for a larger sample of 157 grasses including one C_3_ and one C_4_ *A. semialata* and representing 17 independent C_4_ lineages. Predictor variables in this analysis were based on the anatomical measurements of Christin *et al*. (2013) and chosen to best match the variables used in the *A. semialata* LDA described above, including the number of mesophyll cells between veins, mesophyll cell width, proportion of veins that are minor, leaf thickness, inner BS cell width and outer BS cell width. The species were grouped as C_3_, C_4_ species using the inner BS and C_4_ species using the outer BS.

### Vein order analysis

To determine whether the pattern of vein density observed in the main data set was maintained across a larger sample of *A. semialata*, we counted the total number of veins per segment and the presence or absence of minor veins in 91 additional accessions consisting of herbarium specimens that had been rehydrated in distilled water overnight at 4°C prior to embedding, sectioning, staining and imaging as described above. Together with the 50 previous samples, this larger data set included a total of 72 C_4_ (i.e. δ^13^C > −17‰) and 69 non‐C_4_ (i.e., δ^13^C < −17‰) accessions distributed across the species’ geographic range.

### Ultrastructure and immunohistochemistry

To investigate whether ultrastructural changes might differ between photosynthetic types within this species, we analysed the spatial distributions of organelles and enzymes in one population representing each of the C_3_, C_3_‐C_4_ and C_4_ types. Recently expanded mature leaf tissue was prepared for transmission electron microscopy and processed for immunodetection of the large subunit of Rubisco (RBCL) and glycine decarboxylase H subunit (GLDH) as previously described (see Supporting Information Materials 1; Khoshravesh *et al*. 2017).

### Leaf anatomy in a common environment

To determine the degree to which the various leaf anatomical phenotypes arose from plastic development responses to their differing native growth environments, we compared field phenotypes to those obtained from live tillers of 17 *A. semialata* accessions (5 C_3_, 4 C_3_‐C_4_ and 8 C_4_) after growing for a minimum of 3 months in a common growth chamber, with conditions as described in Lundgren *et al*. 2016. The environmental conditions (Fick & Hijmans 2017) at the field collection sites are detailed in Table S2. On controlled environment samples, the number and order of veins, minimum number of M cells separating veins, area of inner BS cells, segment length and thickness, and IVD were determined on one segment per leaf.

### Plasticity for leaf anatomy in response to low CO_2_

To further test whether C_4_‐compatible phenotypes could emerge from plastic responses to the environment, as previously suggested (Li *et al*. 2014), we carried out a CO_2_ manipulation experiment designed to promote photorespiration. One C_4_ (MDG, South Africa) and one C_3_ (GMT, South Africa) plant were initially grown from seed in a controlled environment chamber set as described in Lundgren *et al*. 2016, but with 400 μmol mol^−1^ CO_2_ concentration. Both plants were split into five replicate cuttings and kept in the same growth chamber conditions to re‐establish for four months. From each replicated clone, one fully expanded, mature leaf was sampled and fixed in 4 : 1 ethanol : acetic acid solution. The growth chamber was then set to 180 μmol mol^−1^ CO_2_ concentration for the next four months to promote photorespiration, while maintaining the other environmental conditions, and one new fully expanded leaf was again sampled and fixed. All leaf samples from the 400 (i.e. ambient) and 180 (i.e. low) CO_2_ treatments were embedded, sectioned and imaged as described above. To determine whether the plants used different photosynthetic pathways in the two CO_2_ growth environments, we determined CCP and carboxylation efficiency, as described in Lundgren *et al*. (2016).

## Results

### *Alloteropsis semialata* presents a continuum of leaf anatomy

**Figure 2:**
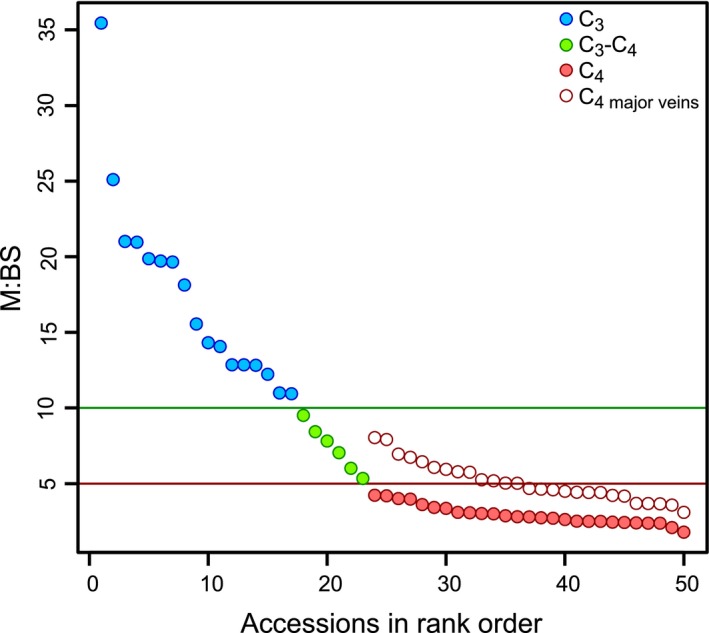
Continuous variation in *Alloteropsis semialata* leaf anatomy, but distinct division among C_3_, C_3_‐C_4_ and C_4_ types. Ratios of mesophyll (M) to bundle sheath (BS) area of individual accessions of C_3_ (blue circles), C_3_‐C_4_ (green circles) and C_4_ (solid red circles) plants, ranked by M:BS value. *n* = 50. Lines delineating M:BS ratios that distinguish C_3_ from C_3_‐C_4_ (green) and C_3_‐C_4_ from C_4_ (red) are shown. For C_4_ individuals, M:BS ratios are also calculated in the absence of minor veins (open red circles).

The ratio of M to BS tissue, a trait known to differ among C_3_ and C_4_ species (Hattersley 1984), forms a continuum within *Alloteropsis semialata*, along which photosynthetic types are sorted (Fig. 2). Indeed, the smallest values are restricted to C_4_ accessions and the largest are found in C_3_ individuals. When considering the area in cross section between two secondary veins (i.e., a leaf segment from here onwards; Fig. S1), the M area is over 10 times larger than the BS area in C_3_ accessions, but less than five times larger in C_4_ accessions (Data set S1). As expected, C_3_‐C_4_ accessions are intermediate in their overall leaf anatomy, with 5–10 times more M than BS. These M : BS ranges are consistent with those measured in other C_3_ and C_4_ grasses (Christin *et al*. 2013).

### C_3_, C_3_‐C_4_ and C_4_ *Alloteropsis semialata* have distinct leaf anatomy

**Figure 3:**
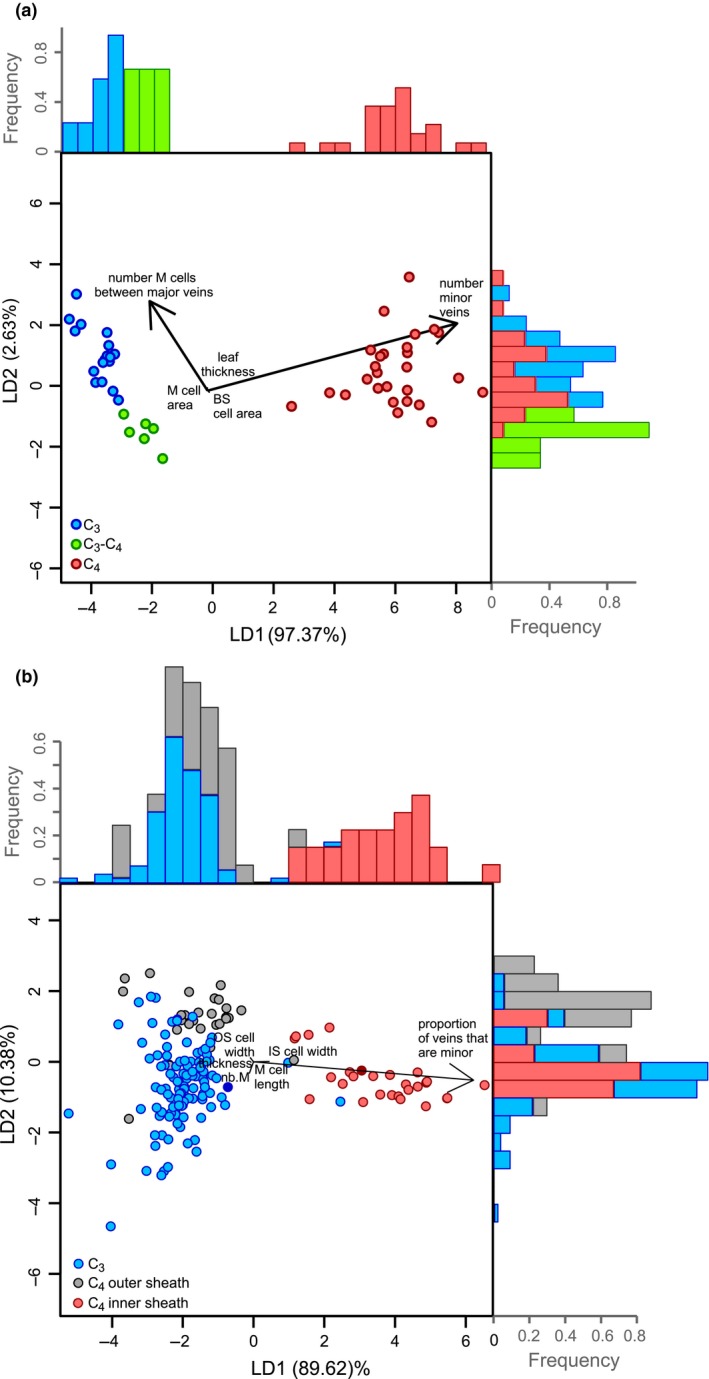
Linear discriminant analysis of leaf anatomical traits. The first (LD1) and second (LD2) dimensions of the LDA are plotted against each other with histograms of each dimension shown on the opposing axis for (a) the LDA on C_3_, C_3_‐C_4_ and C_4_ *Alloteropsis semialata* accessions and (b) the LDA on 157 C_3_, C_4_ inner sheath and C_4_ outer sheath grass species. In addition, one C_3_ and one C_4_ *A. semialata* accession were included in this larger LDA and are denoted by solid blue and red circles respectively. Loading plots are overlaid via black arrows. M, mesophyll; IS, inner sheath; OS, outer sheath; nb.M, number of mesophyll cells between veins.

Variation in M : BS ratios can arise via changes to several underlying traits (Lundgren *et al*. 2014). Our modelling shows that M area is the product of leaf thickness and interveinal distance (IVD; Table 1). The latter is predicted by the number and size of M cells between veins (Table 1). BS area is explained by the number of BS units (i.e. the number of veins per segment) and the size of BS cells (Table 1). When these potential explanatory traits are incorporated within an LDA, all variance between the three photosynthetic types is captured (Fig. 3a). In a bootstrapped sample, the mean overall predictive accuracy is 0.986, which is statistically indistinguishable from 1.0 (95% CI = 0.966–1.000). The mean predictive accuracy for C_4_ (0.999, 95% CI = 0.995–1.000), C_3_ (0.976, 95% CI = 0.918–1.000) and C_3_‐C_4_ (0.926, 95% CI = 0.805–1.000) accessions is also statistically indistinguishable from 1.0. The analysis, therefore, confirms that leaf anatomy varies among photosynthetic types in a statistically predictable manner.

**Table 1:** Results of linear regression analyses on leaf components underlying M : BS ratios in *Alloteropsis semialata*

	*F*	df	Adj *R* ^2^	*P*	*t*	*P*
Total M area/segment (μm^2^)	59.35	2, 47	0.704	1.38 × 10^−13^		
Leaf thickness (μm)					3.98	0.00024
Interveinal distance* (μm)					10.58	5.07 × 10^−14^
Interveinal distance (μm)	343.4	2, 47	0.933	< 2.2 × 10^−16^		
Number M cells between veins†					25.48	< 2 × 10^−16^
M cell size (μm^2^)					6.97	9.22 × 10^−9^
Total BS area/segment (μm^2^)	124.8	2, 47	0.835	< 2.2 × 10^−16^		
BS cell size‡ (μm^2^)					9.52	1.54 × 10^−12^
Vein density (veins/segment)					4.23	0.00011

*Average distance between the center points of all veins.

†Number of mesophyll (M) cells between all veins.

‡Cross‐sectional area of inner bundle sheath (BS) cells on tertiary order veins.

The first axis of the LDA explains 97.37% of the variance between photosynthetic types and clearly distinguishes C_4_ from non‐C_4_ accessions (Fig. 3a). This axis is most strongly associated with the number of minor veins per segment, which were absent from all non‐C_4_ accessions in this analysis (Table 2). The second axis explains 2.63% of the variance between groups, clearly distinguishes C_3_ from C_3_‐C_4_ plants, and is most strongly associated with the number of M cells between major veins (i.e. secondary and tertiary order veins) and the number of minor veins (Fig. 3a; Table 2). Since minor veins are restricted to C_4_ individuals, their contribution to LD2 is linked to diversity within the C_4_ group. These results indicate that most of the variance in the data set stems from the contrast between C_4_ and non‐C_4_ individuals and is driven entirely by a single underlying trait, the presence of minor veins. The phenotypic distance between C_3_ and C_3_‐C_4_ individuals is very small, being explained by the number of M cells between major veins. Conversely, leaf thickness and the cross‐sectional areas of individual BS and M cells poorly distinguish photosynthetic types in this species.

**Table 2:** Coefficients of linear discriminants in a linear discriminant analysis on (top) five leaf anatomical traits expected to drive overall mesophyll to bundle sheath area ratios in *Alloteropsis semialata* and on (bottom) six leaf anatomical traits in 157 grass species, grouped as C_3_ species, C_4_ species using the inner sheath and C_4_ species using the outer sheath

LDA on *Alloteropsis semialata* accessions		
Trait	LD1	LD2
Number of minor veins per segment	1.3591	0.3663
Number of mesophyll cells between major veins	−0.315	0.4881
Average area inner bundle sheath cell on tertiary veins (μm^2^)	0.0123	−0.0104
Average area mesophyll cell (μm^2^)	−0.0012	−7.82E‐05
Leaf thickness (μm)	−5.52E‐04	0.0189

LDA on 157 grass species + 1 C_3_ and 1 C_4_ *Alloteropsis semialata* accession		
Trait	LD1	LD2
Proportion of veins that are minor	4.145779	−0.34605
Outer bundle sheath (BS) cell width (μm)	−0.06433	0.080823
Inner BS cell width (μm)	0.301257	0.004749
Leaf thickness (μm)	−0.0083	−0.00729
Mesophyll cell width (μm)	0.01989	−0.01048
Number of mesophyll cells between veins	−0.08007	−0.22237

The first two axes of an LDA of anatomical traits on the larger species data set explain 89.62% and 10.38% of the variation respectively. The first axis clearly distinguishes C_4_ species that use the inner bundle sheath from C_4_ species using the outer sheath and the C_3_ species (Fig. 3b) and is mostly associated with the proportion of minor veins, while the remaining anatomical traits are weakly correlated with both axes (Table 2).

### Differences between C_4_ and non‐C_4_ phenotypes arise from the development of minor veins

**Figure 4:**
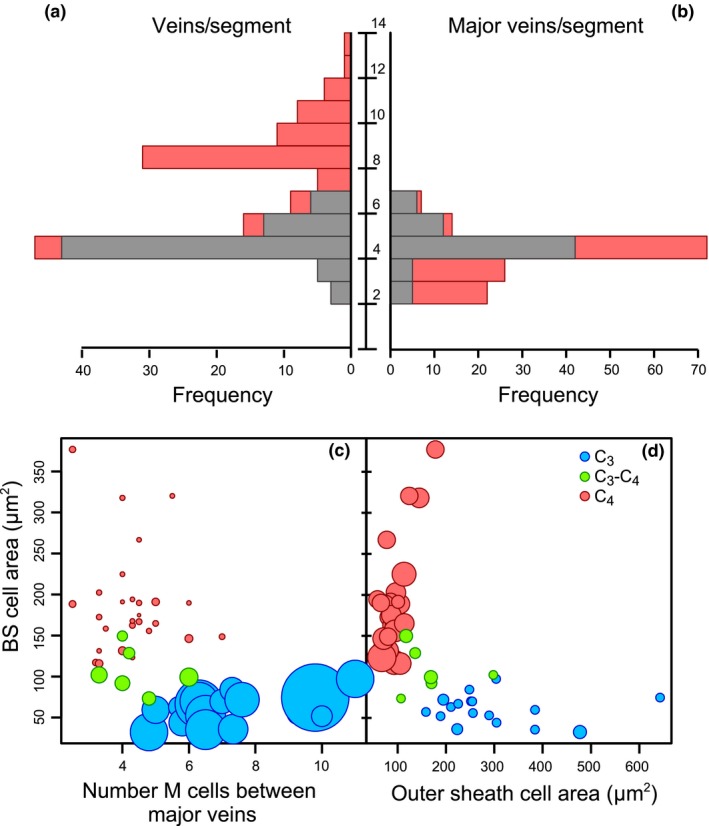
Diversity of intraspecific anatomical components. Histograms of (a) vein density (i.e., the total number of veins per segment) and (b) the number of major veins per segment in C_4_ (red; *n* = 72) and non‐C_4_ (grey, *n* = 69) accessions. Scatter plots show (c) the average number of mesophyll (M) cells between major veins vs. the average area of individual bundle sheath (BS) cells, with dot size proportional to the M:BS ratio, and (d) BS cell area vs. outer sheath cell area, with dot size proportional to the number of veins per segment. Colours indicate photosynthetic type with C_3_ (blue; *n* = 17), C_3_‐C_4_ (green; *n* = 6) and C_4_ (red; *n* = 27).

In *A. semialata*, the presence of minor veins is the only variable consistently distinguishing C_4_ and non‐C_4_ accessions. When the M : BS ratio is calculated in the absence of minor veins, the clear distinction between C_3_‐C_4_ and C_4_ accessions disappears, with nearly half the C_4_ accessions overlapping with C_3_‐C_4_ plants (Fig. 2). This shows that the development of minor veins in C_4_ accessions reduces the M : BS ratio by increasing BS area and displacing M area. To confirm the restriction of minor veins to C_4_ individuals, we screened vein architecture in a larger data set of *A. semialata* (Fig. 4a,b; Data set S2). Minor veins were present in all C_4_ accessions and absent in all but five non‐C_4_ accessions. Four of these had only occasional and irregularly spaced minor veins, while the final accession is an individual originating from a natural cross between C_3_‐C_4_ and C_4_ individuals (Olofsson *et al*. 2016). Our data therefore show that the presence of frequent and regularly spaced minor veins is universally and uniquely associated with the C_4_ genomic background, captures nearly all of the anatomical variation between C_4_ and non‐C_4_ phenotypes and explains overall differences in relative M and BS areas.

The proliferation of minor veins explains a number of patterns associated with C_4_ anatomy. As expected, the number of M cells between consecutive veins differs among photosynthetic types, being the smallest in C_4_ accessions (1–3), compared to C_3_‐C_4_ (3–6) and C_3_ (5–11) plants (Fig. S2). However, the number of M cells between major veins overlaps between the C_4_ and non‐C_4_ groups (Fig. 4c), which indicates that the reduced distance between any pair of M and BS cells in C_4_ accessions is caused by the differentiation of ground meristem cells into minor veins rather than a reduced proliferation of M cells. The high vein density of C_4_ plants following the development of minor veins is accompanied by more than a twofold increase in extraxylary fibres (i.e. tissue area per segment length) than is found in non‐C_4_ accessions (Fig. S3). As the area of extraxylary fibres per vein does not differ between the photosynthetic types (Fig. S3), the increased fibre area in C_4_ plants derives entirely from their greater vein density.

### Other anatomical changes happened before or after the transition from C_3_‐C_4_ to C_4_ physiology

The development of minor veins explains the overall anatomical difference between C_4_ and non‐C_4_ accessions and is therefore linked to the emergence of a fully functioning C_4_ physiology from a C_3_‐C_4_ intermediate state. Evolutionary changes that happened once this C_4_ physiology was in place would be restricted to some, but not all, C_4_ individuals. In our data set, such changes include further reductions to the M : BS ratio, potentially achieved via contractions to M airspace (Byott 1976) or increases in BS cell size. Indeed, although BS cell sizes of different photosynthetic types overlap, large increases to BS cell size characterise some African C_4_ accessions (Figs S4 and S5). The BS cell enlargement was therefore involved in the adaptation of C_4_ physiology after it had emerged, possibly to accommodate more or larger organelles for a more efficient C_4_ cycle, rather than being involved in its origin. Occasional hybridisation between C_4_ and non‐C_4_ individuals could affect the distribution of trait values; however, non‐C_4_ *A. semialata* individuals are restricted to Africa, so that hybridisation outside of Africa is unlikely. Yet, Asia and Australian accessions exhibit some of the smallest BS cells among C_4_ accessions (Fig. S5).

Some characters observed in C_4_ accessions are also present in C_3_‐C_4_ individuals, but not C_3_ ones, indicating that they are not associated with the transition to fully functional C_4_ physiology, but might have facilitated it. These include a small increase in BS cell sizes in C_3_‐C_4_ compared with C_3_ plants and a decrease in outer sheath cell size, with C_3_‐C_4_ accessions bridging the anatomical gap between C_3_ and C_4_ outer sheath cell sizes (Fig. 4c,d). This reduced outer sheath in C_3_‐C_4_ and C_4_ *A. semialata* likely facilitates metabolite exchanges between M and BS cells.

### Differences between C_4_ and non‐C_4_ leaves are not environmentally induced

*Alloteropsis semialata* plants grow naturally in diverse environments, depending on their photosynthetic background and evolutionary history (Lundgren *et al*. 2015). To verify that the differences we observe among photosynthetic types are not induced by environmental variations, we compared the leaves of field‐collected plants after transplanting and growing them in a common controlled environment growth chamber for at least 3 months (Data set S3; Fig. S6). Compared to field conditions, C_3_ accessions produced more M cells between veins (*P *=* *0.044) in the common environment. Moreover, C_3_‐C_4_ plants produced thicker leaves (*P *=* *0.040), such that leaf thickness of the three photosynthetic types converged in the common environment, which is likely a result of the non‐limiting light, nutrients and water available in these conditions. However, the other traits were not influenced by growth conditions and leaf anatomy of the three photosynthetic types remained distinct when grown in the common environment.

We further verified that historical changes in atmospheric composition did not influence the leaf phenotype by comparing C_3_ and C_4_ *A. semialata* under current ambient (400 ppm) and the Pleistocene minimum (180 ppm) CO_2_ concentrations. Plants grown under the low CO_2_ concentration experience elevated photorespiration rates, which might have induced a more C_4_‐like anatomy. However, we found that plants did not shift photosynthetic state under the differing CO_2_ conditions (i.e. mean CCPs in ambient/low CO_2_ for C_3_ = 49.8/53.1 and C_4_ = 4.6/8.1 μmol mol^−^¹; Data set S4). Both C_3_ and C_4_ plants produced thinner leaves in the low CO_2_ environment (*P *=* *0.0049 C_3_/0.0065 C_4_), and C_4_ plants developed smaller BS cells (*P *=* *0.011; Fig. S6), probably because the lower carbon supply restricted development (Ripley *et al*. 2013). Importantly, the C_3_ plants did not produce more veins (or any minor veins), larger BS cells or fewer M cells between veins when grown under this high photorespiration condition. These results show that, even when photorespiration is high, a C_4_‐like phenotype is not plastically induced in C_3_ *A. semialata*.

## Discussion

Photosynthetic types form a continuum, along which multiple biochemical, anatomical and ultrastructural alterations increase the proportion of CO_2_ fixed via the C_4_ cycle. The emerging model of C_4_ evolution involves gradual and overlapping phenotypic changes (Heckmann *et al*. 2013; Sage *et al*. 2014; Bräutigam & Gowik 2016; Schlüter & Weber 2016; Dunning *et al*. 2017), with traits acquired in differing orders among C_4_ lineages (Williams *et al*. 2013). Different traits may be involved in the initial transition to a C_4_ phenotype and the subsequent adaptation and diversification of that phenotype (Christin & Osborne 2014; Watcharamongkol *et al*. 2018). Within the grass *Alloteropsis semialata*, we have shown that the only gross leaf property distinguishing all C_4_ from all non‐C_4_ phenotypes is the development of frequent minor veins. The presence of these minor veins has multiple consequences, including an overall increase in vein density, enlargement of the total volumes of BS tissue and a displacement of M tissue. These anatomical changes combine to facilitate C_4_ cycle activity, as demonstrated by a strong correlation between leaf vein frequency and carbon isotope composition observed for this species (Lundgren *et al*. 2016). Our analyses of leaf ultrastructure indicate that the evolution of C_4_ photosynthesis in *A. semialata* may have involved additional changes in organelle distribution among cell types (Supporting Information Materials 1; Figs S7–S9), although the small sample of populations prevents us from differentiating ultrastructural changes linked to the transition to C_4_ from those that happened later.

The change in venation inferred during the evolution of C_4_ photosynthesis in *A. semialata* may have a number of physiological and ecological consequences. First, the increase in vein frequency is accompanied by an enhancement of unpigmented extraxylary fibres, which improves light transmission to the BS, and thus ATP production in these cells, facilitating photosynthetic carbon reduction (Bellasio & Lundgren 2016). Enhanced fibre density may also increase leaf toughness, reduce digestibility and consequently deter herbivores (Caswell *et al*. 1973; Wilson *et al*. 1983). Secondly, the insertion of additional veins may influence leaf hydraulics. Model simulations for other plant species demonstrate that an increase in minor vein density can lead to greater leaf hydraulic conductance (McKown *et al*. 2010). However, empirical studies show that this is unlikely to improve drought tolerance, since the decline in hydraulic conductance during drought arises primarily outside veins (Scoffoni & Sack 2017; Scoffoni *et al*. 2017a), while embolisms arise first in the midrib, not minor veins (Scoffoni *et al*. 2017b).

Our results complement those from previous comparisons among species, which show that an additional order of minor veins develops during the evolutionary transition from non‐C_4_ to C_4_ forms of *Flaveria* (McKown & Dengler 2009), while BS cell size is large in both C_3_ and C_4_ *Flaveria* species (Kümpers *et al*. 2017). Our further analysis of leaf gross anatomy across multiple grass species shows that the insertion of additional minor veins is a frequent developmental mechanism for decreasing the M : BS ratio in those C_4_ grasses that primarily localise Rubisco within the mestome sheath. The insertion of minor veins could occur via relatively few developmental changes, likely underpinned by changes to auxin, brassinosteroids, SHORTROOT/SCARECROW and/or INDETERMINATE DOMAIN transcription factors (Kumar & Kellogg 2018; Sedelnikova *et al*. 2018). In grasses, vein orders develop sequentially as leaves grow wider, such that minor veins are initiated considerably later than other vein orders, usually once the leaf ceases to widen (Nelson & Langdale 1989; Sedelnikova *et al*. 2018). Thus, the development of functional minor veins likely arises via the heterochronic regulation of the existing machinery for vein formation, sustaining vein differentiation beyond that of non‐C_4_ plants (Nelson 2011; Sedelnikova *et al*. 2018), probably through the prolonged production of auxin during later phases of leaf elongation (Scarpella *et al*. 2010). Alternatively, minor veins may also result from a heterotopic specialisation of auxin maxima that permits them to form closer together (Kumar & Kellogg 2018).

The possibility that a transition from non‐C_4_ to C_4_ states can be caused by a single developmental alteration is a plausible explanation for the recurrent origins of C_4_ leaf anatomy and helps to resolve the paradox of how this complex trait emerged so many times. We also show that organelle number and size differ among photosynthetic types of *A. semialata*, but, here too, recent work indicates that one gene can control multiple ultrastructural modifications (Wang *et al*. 2017). Finally, transcriptome comparisons show that few genes encoding enzymes are upregulated during the transition from non‐C_4_ to C_4_ in *A. semialata* (Dunning *et al*. 2017). We therefore conclude that the overall transition from a non‐C_4_ state to the form of C_4_ photosynthesis observed in *A. semialata* involved relatively few genetic mutations.

The limited number of changes involved in the emergence of C_4_ anatomy in *A. semialata* is partially explained by the presence of relatively enlarged BS in the C_3_‐C_4_ accessions, while C_3_ *A. semialata* BS size is similar to C_3_ species from other grass lineages (Lundgren *et al*. 2014, 2016; Dunning *et al*. 2017). C_3_‐C_4_ *A. semialata* are also characterised by fewer M cells compared to C_3_ accessions and higher BS organelle abundance (Figs 3a, 4c and S7–S9). These properties that had been selected for the C_3_‐C_4_ physiology eased the subsequent transition to a full C_4_ state, but it is important to note that the physiology and anatomy of C_3_‐C_4_ *A. semialata* are typical for C_3_‐C_4_ plants in general (Lundgren *et al*. 2016), and their anatomical characteristics can be found among C_3_ grasses (Hattersley 1984; Christin *et al*. 2013; Lundgren *et al*. 2014). The background against which C_4_ anatomy evolved in *A. semialata* is therefore not exceptional.

Our conclusion that C_4_ leaf anatomy can arise from one key developmental modification is apparently incompatible with the great anatomical specialisation of other C_4_ lineages as well as the large phenotypic gaps separating them from their closest C_3_ relatives (Dengler *et al*. 1994; Christin *et al*. 2013). However, most C_3_ and C_4_ sister lineages are separated by long periods of evolution, and comparing these groups therefore captures all of the changes that happened after the origin of C_4_ photosynthesis to improve the efficiency of C_4_ physiology and adapt it to various organismal and ecological contexts (Christin & Osborne 2014). Indeed, photosynthetic efficiency may be significantly lower in C_4_ *A. semialata* than in species from some older C_4_ lineages (Lundgren *et al*. 2016; Bräutigam *et al*. 2018). This suggests that C_4_ photosynthesis in *A. semialata* may represent a rudimentary version of the physiological trait (Ueno & Sentoku 2006). The biochemical characteristics of the C_4_ cycle in *A. semialata* may be one reason for this (Bräutigam *et al*. 2018), and the presence of Rubisco protein in M could be another (Ueno & Sentoku 2006). Anatomical diversity may also explain some of the variation in physiological efficiency among *A. semialata* populations (Lundgren *et al*. 2016). Indeed, in *A. semialata*, enlargements of the BS cells beyond those seen in non‐C_4_ individuals are restricted to a subset of C_4_ populations (Fig. 2) and thus happened after the emergence of C_4_ physiology. Over time, accumulated modifications will move C_4_ leaf anatomy far beyond that realised via a single developmental change. However, the fact that an initial C_4_ phenotype and the associated physiology can be accessed via a single modification likely placed multiple groups on a selective highway to highly specialised and successful variants of the C_4_ syndrome.

## Authorship

MRL, PAC and CPO designed the study. MRL produced and analysed the data, with the help of LTD, JJMV and JWB. TS and RK performed the immunolocalisations and TEM imaging. SS assisted with immunolocalisation sample preparation. MS assisted with tissue fixation. MRL, LTD, JKO, BR, MSV, GB, CA, NC, AM, MB, CML, PAC and CPO contributed plant material. MRL, PAC and CPO interpreted the results and wrote the paper, with the help of all the authors.

## Data Availability

Data available from the Dryad Digital Repository: https://doi.org/10.5061/dryad.q7r61k7
