# Supplementary material for: C4 anatomy can evolve via a single developmental change
Source: Ecol Lett. 2018 Dec 17;22(2):302–12. doi: 10.1111/ele.13191 (PMC6849723; doi:10.1111/ele.13191)
Supplement: Supplementary file 1 [file ELE-22-302-s001.pdf]

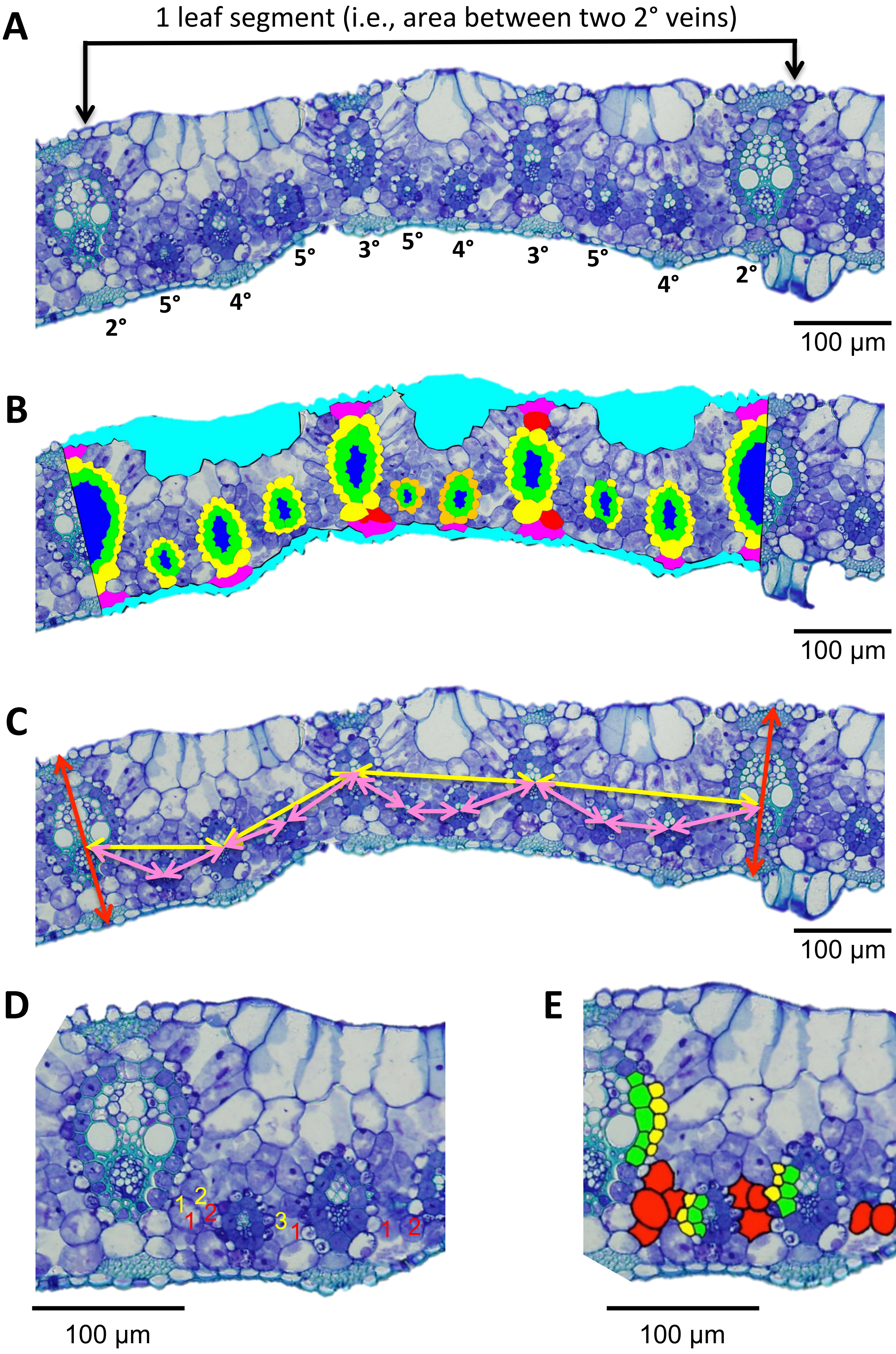

A
1 leaf segment (i.e., area between two 2° veins)
2°
5°
4°
5°
3°
5°
4°
3°
5°
4°
2°
100 µm
B
100 µm
C
100 µm
D
1
2
1
2
3
1
1
2
100 µm
E
100 µm

Figure S1

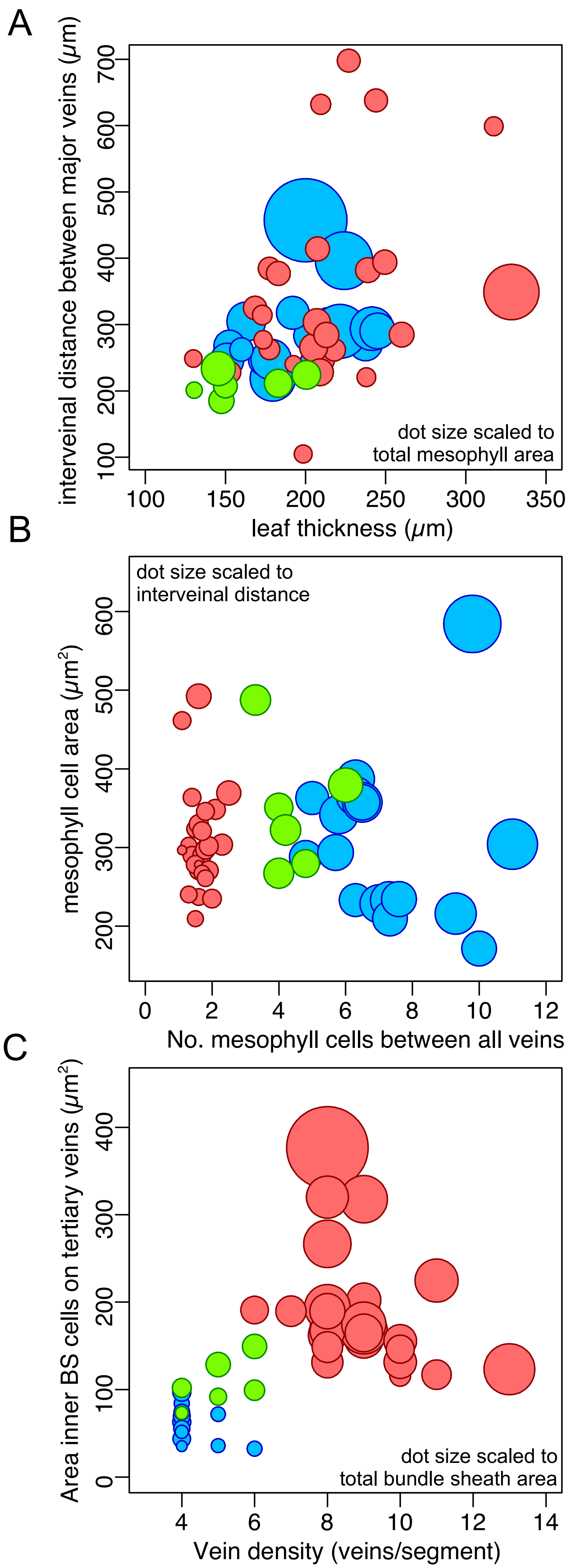

A
interveinal distance between major veins (μm)
100
200
300
400
500
600
700
dot size scaled to
total mesophyll area
100
150
200
250
300
350
leaf thickness (μm)
B
dot size scaled to
interveinal distance
mesophyll cell area (μm^2)
200
300
400
500
600
0
2
4
6
8
10
12
No. mesophyll cells between all veins
C
Area inner BS cells on tertiary veins (μm^2)
0
100
200
300
400
dot size scaled to
total bundle sheath area
4
6
8
10
12
14
Vein density (veins/segment)

Figure S2

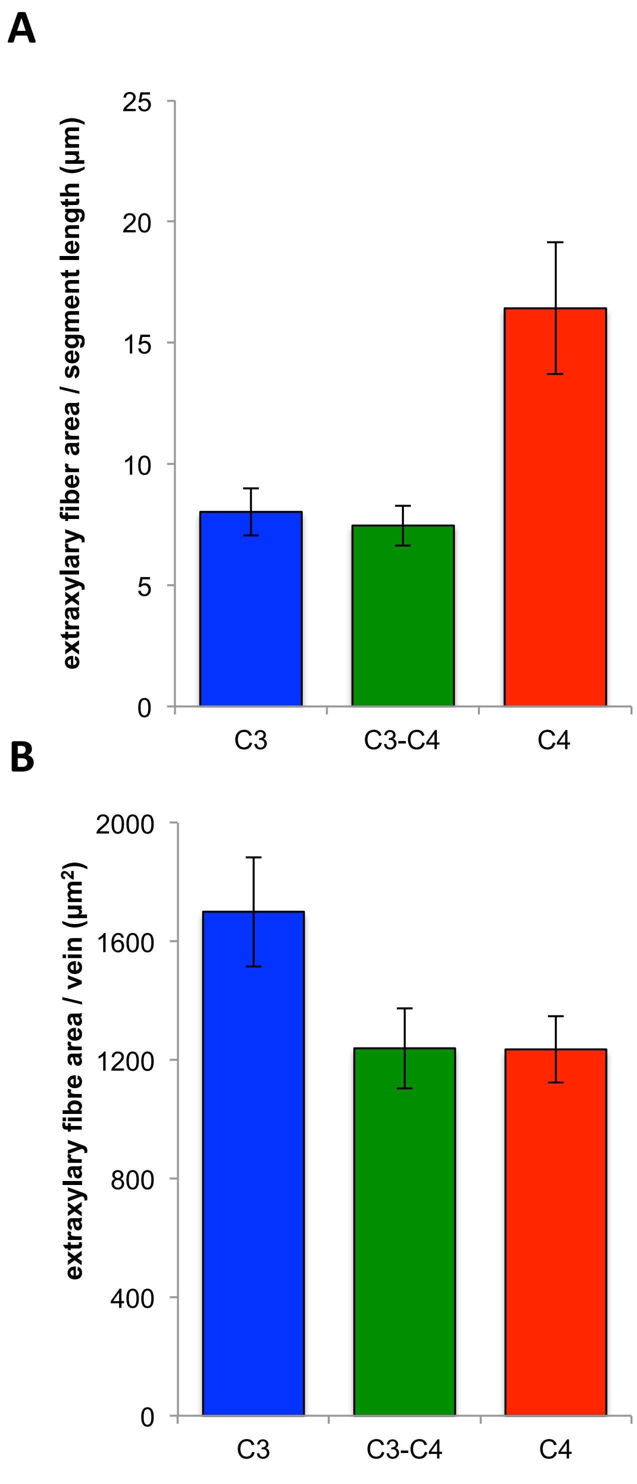
A
extraxylary fiber area / segment length (µm)
25
20
15
10
5
0
C3
C3-C4
C4
B
extraxylary fibre area / vein (µm²)
2000
1600
1200
800
400
0
C3
C3-C4
C4

Figure S3

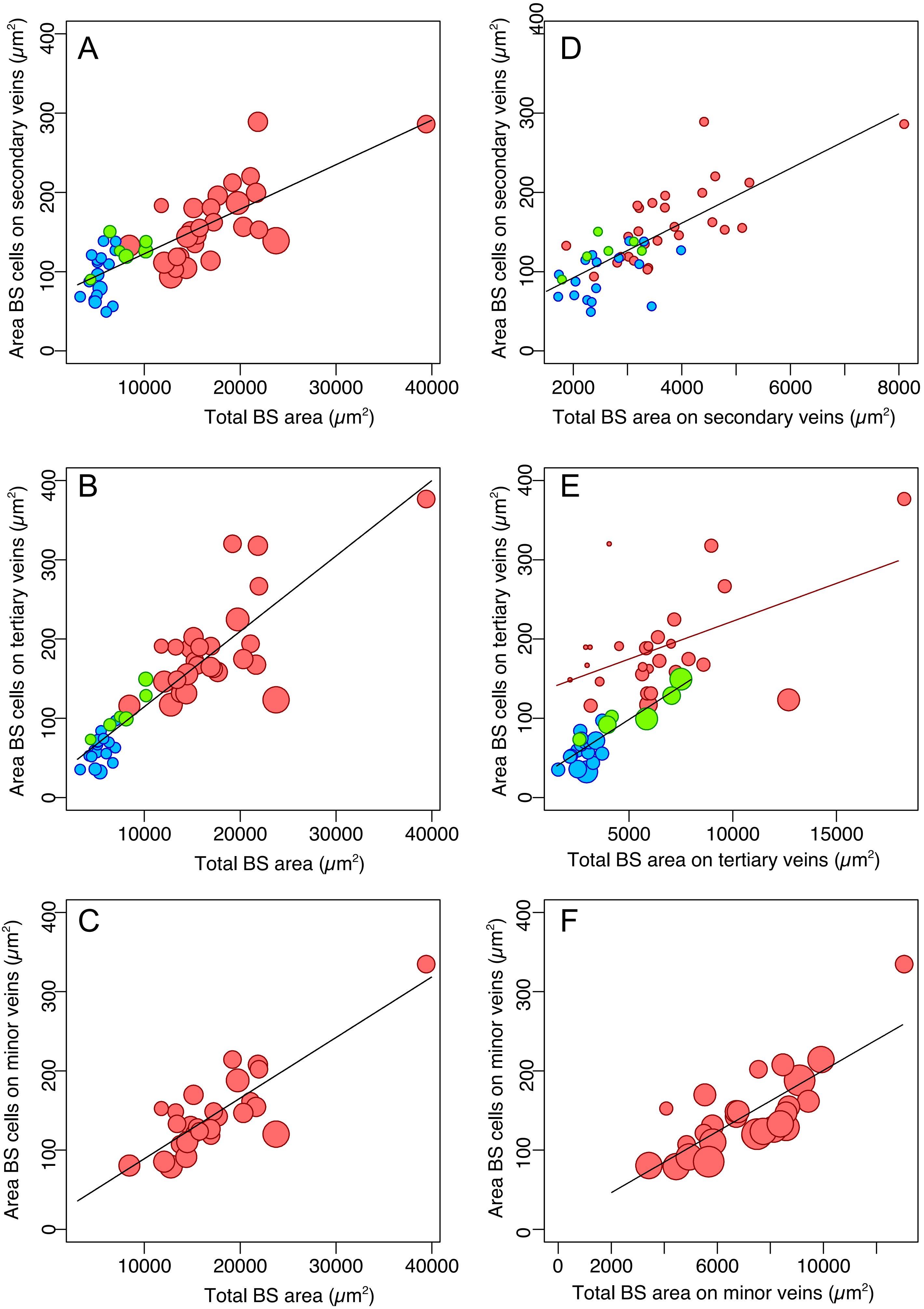

A
B
C
D
E
F
Area BS cells on secondary veins (μm²)
Area BS cells on tertiary veins (μm²)
Area BS cells on minor veins (μm²)
Total BS area (μm²)
Total BS area on secondary veins (μm²)
Total BS area on tertiary veins (μm²)
Total BS area on minor veins (μm²)
0
100
200
300
400
10000
20000
30000
40000
2000
4000
6000
8000
5000
15000

Figure S4

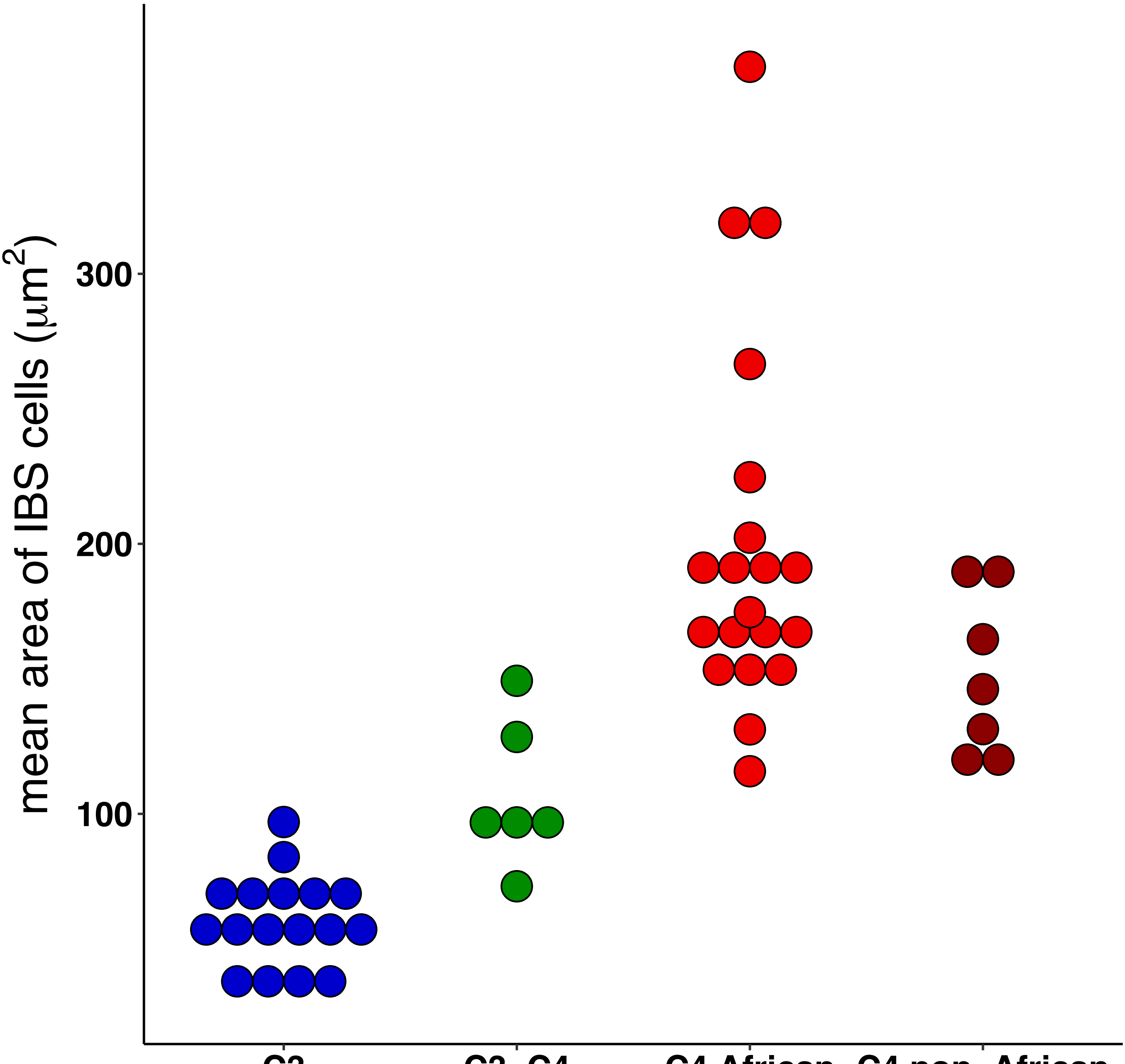
mean area of IBS cells (μm^2)
300
200
100
C3
C3−C4
C4 African
C4 non−African

Figure S5

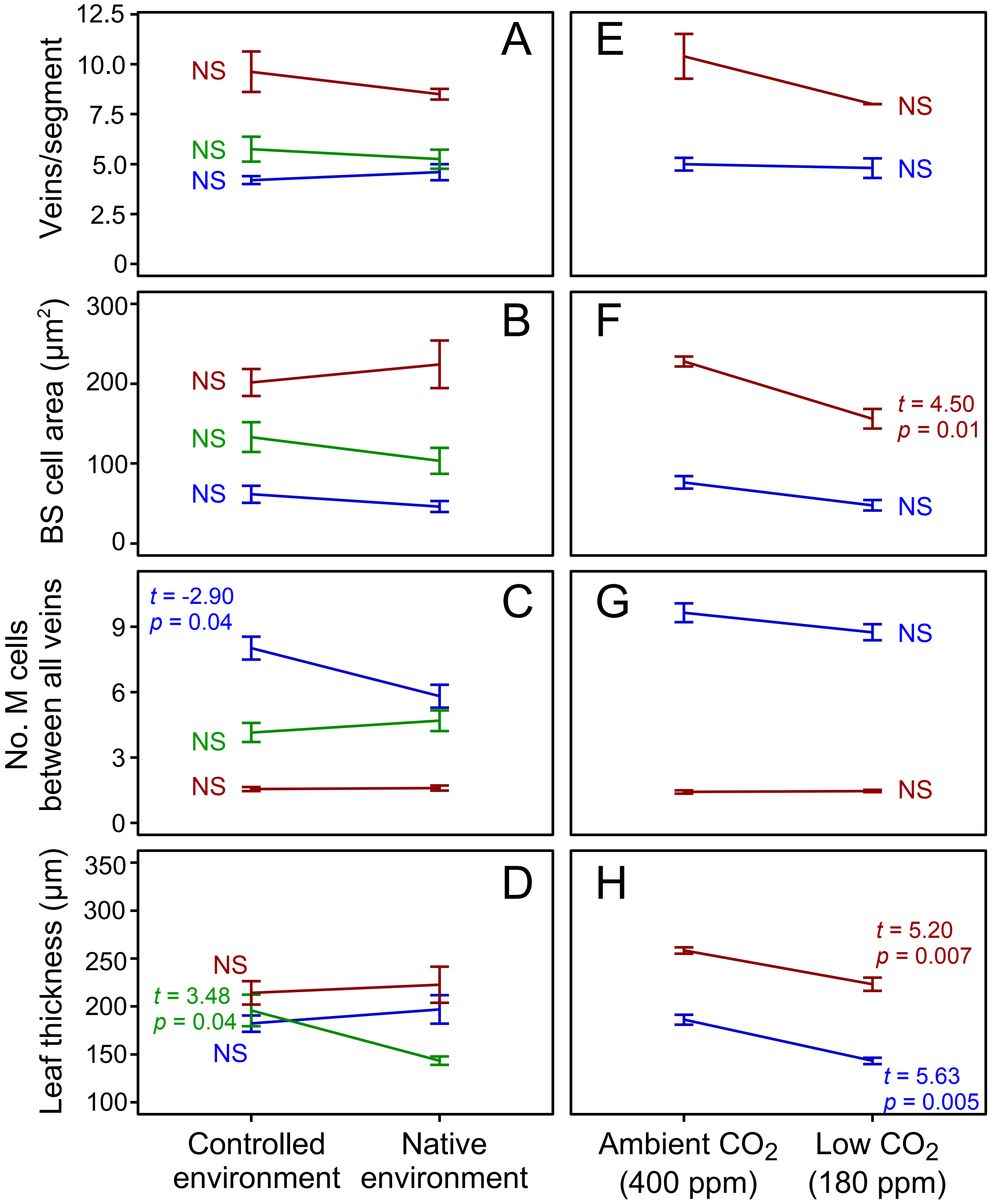
Veins/segment
BS cell area (μm2)
No. M cells between all veins
Leaf thickness (μm)
A
B
C
D
E
F
G
H
NS
t = 4.50
p = 0.01
t = -2.90
p = 0.04
t = 3.48
p = 0.04
t = 5.20
p = 0.007
t = 5.63
p = 0.005
Controlled environment
Native environment
Ambient CO2 (400 ppm)
Low CO2 (180 ppm)

Figure S6

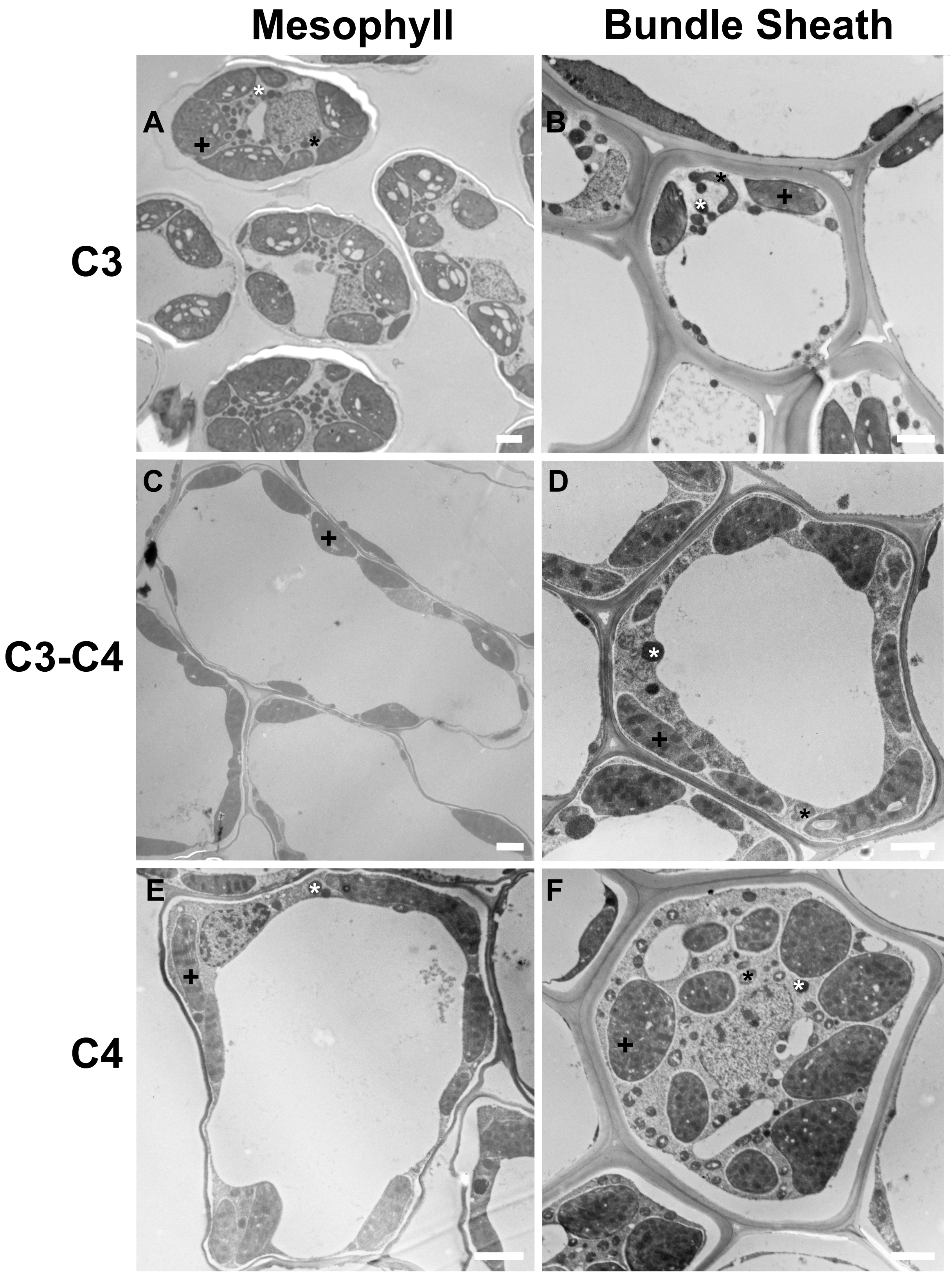

Figure S7

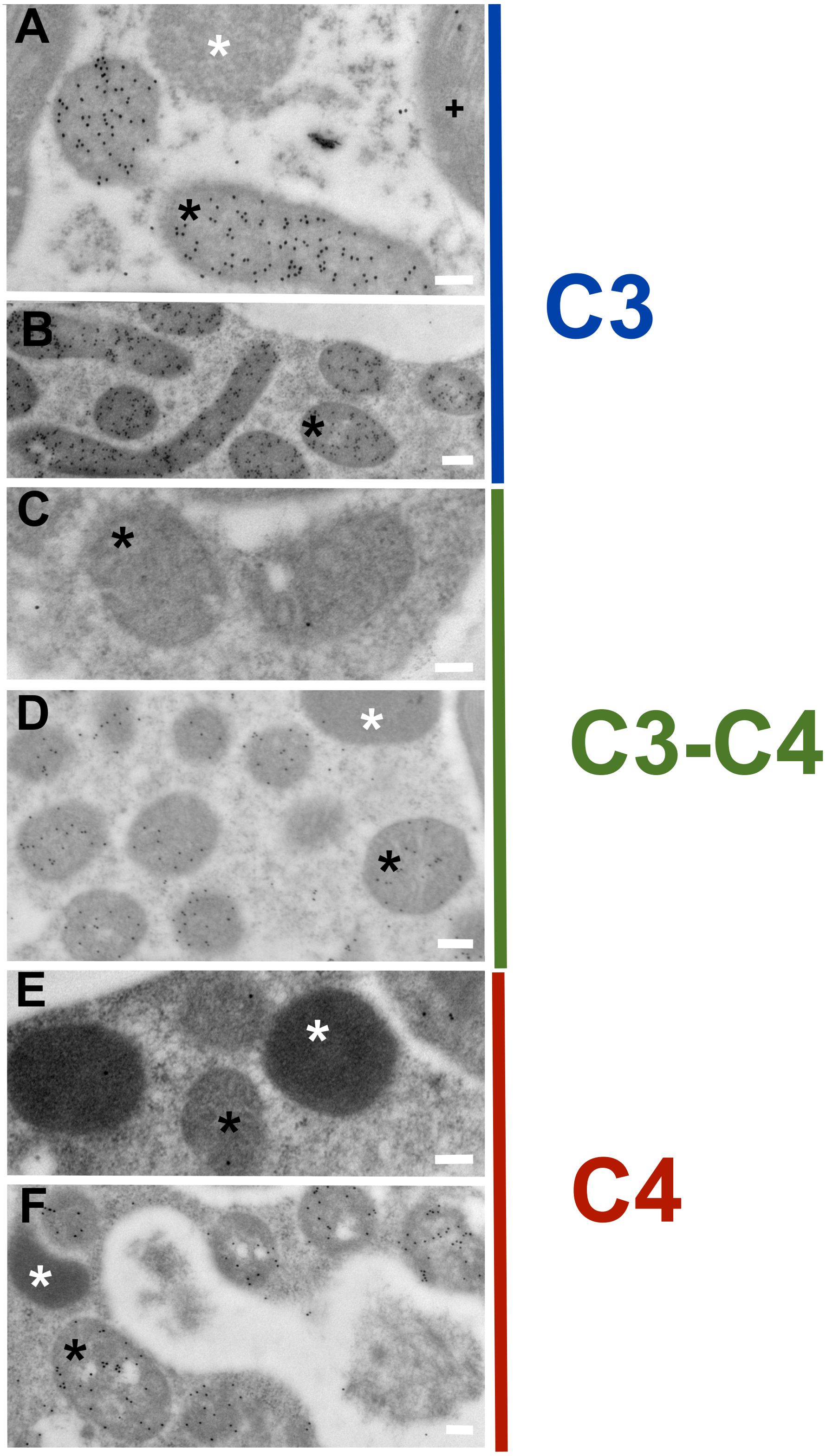

Figure S8

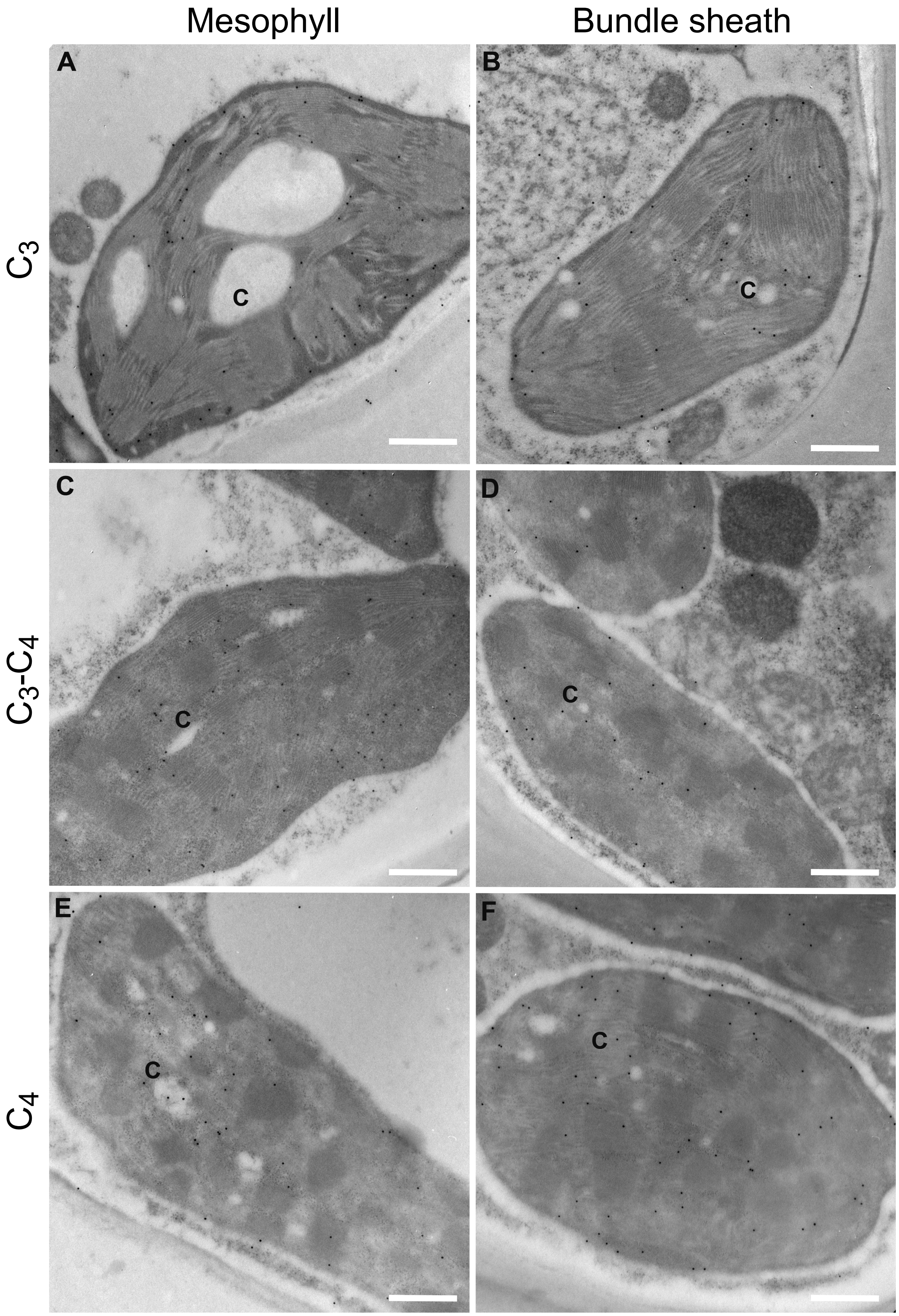

Figure S9
