# Supplementary material for: C4 anatomy can evolve via a single developmental change
Source: Ecol Lett. 2018 Dec 17;22(2):302–12. doi: 10.1111/ele.13191 (PMC6849723; doi:10.1111/ele.13191)
Supplement: Supplementary file 3 [file ELE-22-302-s003.pdf]

SUPPORTING INFORMATION

C_4 anatomy can evolve via a single developmental change

Marjorie R. Lundgren, Luke T. Dunning, Jill K. Olofsson, Jose J. Moreno-Villena, Jacques W. Bouvier, Tammy Sage, Roxana Khoshravesh, Stefanie Sultmanis, Matt Stata, Brad Ripley, Maria S. Vorontsova, Guillaume Besnard, Claire Adams, Nicholas Cuff, Anthony Mapaura, Matheus Bianconi, Christine M. Long, Pascal-Antoine Christin, Colin P. Osborne

SUPPORTING INFORMATION

Supporting Information Materials 1. Ultrastructural characterization of C_3, C_3-C_4 and C_4 accessions

Dataset S1. Comprehensive anatomical measurements for all accessions

Dataset S2. Larger vein density dataset

Dataset S3. Field v controlled environment plasticity dataset

Dataset S4. Growth CO_2 concentration plasticity dataset

Figure S1. Example leaf anatomy measurement methods

Figure S2. Relationships between anatomical traits

Figure S3. Comparison of extraxylary fibre area in C_3, C_3-C_4 and C_4 accessions

Figure S4. Relationships between inner BS cell size and overall BS areas

Figure S5. Comparison of bundle sheath size in African and non-African C_4 accessions.

Figure S6. Plasticity for leaf anatomical components by photosynthetic type

Figure S7. Organelle abundance differs between photosynthetic types.

Figure S8. Immunodetection of GLDH

Figure S9. Immunodetection of Rubisco large subunit

Table S1. Details used to determine photosynthetic pathway for accessions

Table S2. Details of the accessions used in the plasticity dataset

SUPPORTING INFORMATION MATERIALS 1.

ULTRASTRUCTURAL AND IMMUNOLOGICAL CHARACTERIZATION OF C_3, C_3-C_4 AND C_4 ACCESSIONS

Our broad comparative analysis focused on gross leaf anatomy, capturing differences in tissue volumes and cell numbers and sizes. However, previous work in other species has reported ultrastructural differences between photosynthetic types, involving the distribution and characteristics of organelles and subcellular elements (Hattersley *et al.* 1986; Muhaidat *et al.* 2011; Sage *et al.* 2014; Stata *et al.* 2014, 2016). To evaluate whether gross anatomical changes were sufficient for the transition to C_4 photosynthesis, or whether these ultrastructural components might also have been involved, we applied TEM to a subset of plants representing the photosynthetic diversity in *Alloteropsis semialata*, following Khoshravesh *et al.* 2017.

We examined leaf ultrastructure in one accession each representing the C_3 (KWT, South Africa), C_3-C_4 (L04B, Tanzania), and C_4 (QSLD, Australia) types, but did not sample the diversity of C_4 and C_3-C_4 accessions used in the gross leaf anatomy analysis. As such, we are unable to establish the minimum differences between these phenotypes. However, our results indicate that the ultrastructure of M and BS cells differs between the C_3, C_3-C_4, and C_4 accessions. The M chloroplasts of the C_3 accession are more numerous, larger, and cover the cell periphery more than in C_3-C_4 and C_4 types, which is consistent with findings from other physiologically diverse lineages (Fig. S7; Stata *et al.* 2014; 2016). Mitochondria and peroxisomes are also more abundant in C_3 M cells compared to their C_3-C_4 and C_4 counterparts (Fig. S7). The high organelle abundance in the BS of the C_4 accession co-occurs with a constriction of the vacuole (Fig. S7f). Accompanying the activation of BS as the site of carbon concentration, the numbers of BS chloroplasts, mitochondria, and peroxisomes increase between the C_3 and C_4 accessions, with the C_3-C_4 type showing intermediate abundances (Fig. S7). These findings are consistent with previous comparisons of leaf ultrastructure between the photosynthetic types of *A. semialata* (Ueno & Sentoku 2006; Frean *et al.* 1983) and in the grass

genus *Neurachne* (Hattersley *et al.* 1986). In C_3-C_4 and C_4 *A. semialata*, the BS organelles are not localized centrifugally or centripetally along the cell wall (Fig. S7d,f), but are instead evenly dispersed throughout the BS cells. This lack of patterning in organelle arrangement has been observed before in *A. semialata* (Ueno & Sentoku 2006; Frean *et al.* 1983) and other grass species that use the inner sheath for the Calvin cycle (*e.g., Neurachne minor;* Hattersley *et al.* 1986), and likely arises because the combination of BS wall and outer sheath layer represents a strong barrier to CO_2 diffusion out of the BS, such that BS organelles do not need to congregate along the cell periphery to maintain high functionality (Sage *et al.* 2014). Together, these findings are consistent with the hypothesis that the transition between non-C_4 and C_4 states was accompanied by enrichment of BS organelles that displaced vacuole area in *A. semialata*.

Strong labelling of glycine decarboxylase H sub-protein (GLDH) is detected in the mitochondria of C_3 but not C_3-C_4 or C_4 M cells (Fig. S8a,c,e), and in the BS mitochondria of all analysed plant accessions (Fig. S8b,d,f). The absence of GLDH from the M of C_3-C_4 accessions supports the hypothesis that it operates a C_2 photorespiratory CO_2 pump. The presence of GLDH in the BS of C_3 *A. semialata* may indicate the capacity to use a C_2 pump, as has been proposed in the past (Ueno & Sentoku 2006). However, as measurements of CO_2 compensation point in C_3 *A. semialata* accessions consistently fall within the range of typical C_3 grasses (Ueno & Sentoku 2006; Lundgren *et al.* 2016), any C_2 activity in these plants would be very weak. Immunodetection of the large subunit of Rubisco (RBCL) indicates its presence and similar abundance in both the M and BS cells of C_3, C_3-C_4, and C_4 *A. semialata* (Fig. S9), supporting previous findings in the C_3 and C_4 accessions of this species (Ueno & Sentoku 2006). However, C_4 *A. semialata* have more chloroplasts per BS cell and more numerous BS cells via greater vein density, implying more Rubisco in BS than M cells. Although Rubisco is abundant in the M of C_4 *A. semialata*, these plants have clear C_4 $\delta^{13}C$ isotope values (-12.10 ‰), which indicates that little to no atmospheric CO_2 is directly fixed by M Rubisco. Together, these immunolocalization findings indicate a complete shift in GLDH localization between C_3 and

C_3-C_4 phenotypes, no GLDH shift between C_3-C_4 and C_4 *A. semialata*, and no change in RBCL localization across M and BS chloroplasts in *A. semialata*.

SUPPORTING INFORMATION MATERIALS 1 REFERENCES

Frean, M.L., Ariovich, D. & Cresswell, C.F. (1983). C_3 and C_4 Photosynthetic and anatomical forms of *Alloteropsis semialata* (R. Br.) Hitchcock: 2. A comparative investigation of leaf ultrastructure and distribution of chlorenchyma in the two forms. *Ann Bot* **51**, 811-821.

Hattersley, P.W., Wong, S.C., Perry, S. & Roksandic, Z. (1986). Comparative ultrastructure and gas-exchange characteristics of the C_3-C_4 intermediate *Neurachne minor* S. T. Blake (Poaceae). *Plant Cell Environ* **9**, 217-233.

Khoshravesh, R., Lundsgaard-Nielsen. V., Sultmanis, S. & Sage, T.L. (2017). Light Microscopy, Transmission Electron Microscopy, and Immunohistochemistry Protocols for Studying Photorespiration. *Photorespiration.* Humana Press, New York, USA. pp. 243-270.

Lundgren, M.R., Christin P.A., Gonzalez Escobar, E., Ripley, B.S., Besnard, G., Long, C.M., *et al.* (2016). Evolutionary implications of C_3-C_4 intermediates in the grass *Alloteropsis semialata*. *Plant Cell Environ.,* 39, 1974-1885.

Muhaidat, R., Sage, T.L., Frohlich, M.W., Dengler, N.G. & Sage, R.F. (2011). Characterization of C_3–C_4 intermediate species in the genus *Heliotropium* L. (Boraginaceae): anatomy, ultrastructure and enzyme activity. *Plant Cell Environ.,* 34, 1723-1736.

Sage, R.F., Khoshravesh, R., Sage, T.L. (2014) From proto-Kranz to C_4 Kranz: building the bridge to C_4 photosynthesis. *J Exp. Bot.* **65**, 3341-3356.

Stata, M., Sage, T.L., Rennie, T.D., Khoshravesh, R., Sultmanis, S., Khaikin, Y., *et al.* (2014). Mesophyll cells of C_4 plants have fewer chloroplasts than those of closely related C_3 plants. *Plant Cell Environ.,* 37, 2587-2600.

Stata, M., Sage, T.L., Hoffmann, N., Covshoff, S., Wong, G.K-S., & Sage, R.F. (2016). Mesophyll chloroplast investment in C_3, C_4 and C_2 species of the genus *Flaveria*. *Plant Cell Physiol.,* 57, 904-918.

Ueno, O. & Sentoku, N. (2006). Comparison of leaf structure and photosynthetic characteristics of C_3 and C_4 *Alloteropsis semialata* subspecies. *Plant Cell Environ* **29**, 257-268.

SUPPORTING INFORMATION DATASETS

Dataset S1. Comprehensive anatomical measurements for all accessions

Dataset S2. Larger vein density dataset

Dataset S3. Field *vs* controlled environment plasticity dataset

Dataset S4. Growth CO_2 concentration plasticity dataset

SUPPORTING INFORMATION FIGURES

Figure S1. Examples of comprehensive leaf anatomy measurement methods. Panel A demonstrates a single leaf segment, defined as the leaf area between two secondary (2°) veins in cross section. Vein orders are listed below, showing the two flanking 2° veins and the tertiary (3°), quaternary (4°), and quinary (5°) veins within this segment. Leaf segments near the primary vein, or mid-rib, were not included in this study. Throughout the study, secondary and tertiary veins are considered "major" veins and quaternary and quinary veins are considered "minor" veins. Panel B demonstrates the measurements of epidermis and bulliform cells (cyan), outer bundle sheath (yellow), inner bundle sheath (green), vasculature (blue), extraxylary fibres (magenta), and bundle sheath extensions (red). All intervening area not included in these categories was considered mesophyll (uncoloured). Panel C demonstrates the two leaf thickness measurements (red arrows) along the secondary veins that were averaged to determine leaf thickness for each segment and measurements collected to determine the average interveinal distance between all veins (pink arrows) and major (yellow arrows). Panel D shows how the minimum number of mesophyll cells between all veins (red) and between major veins (yellow) were counted, before generating an average value per leaf segment. Panel E demonstrates measurements of individual mesophyll (red), outer bundle sheath (yellow), and inner bundle sheath (green) cells, which were averaged across the leaf segment. The average individual cell areas of inner and outer bundle sheaths were measured separately in secondary (n=10 cells), tertiary (n=12 cells), and minor vein (n=15 cells) orders. The areas of 15 mesophyll cells distributed across the leaf segment were measured and averaged.

Figure S2. Relationships between anatomical traits. Panel A plots leaf thickness against the average interveinal distance between major veins, with dots scaled to the total area of mesophyll per segment. Panel B plots the average number of mesophyll cells between all veins against the average area of individual mesophyll cells, with dots scaled to the average

interveinal distance between all veins. Panel C plots vein density against the average area of individual inner bundle sheath (BS) cells on tertiary veins, with dots scaled to the total area of inner BS per segment. For all plots, C_3 accessions are in blue, C_3-C_4 in green, and C_4 in red.

Figure S3. Comparison of extraxylary fibre area in C_3, C_3-C_4 and C_4 accessions. Comparisons of (A) extraxylary fibre area per segment and (B) extraxylary fibre area per vein. Colors indicate photosynthetic type with C_3 (blue; n=17), C_3-C_4 (green; n=6), and C_4 (red; n=27). Data are mean ± SE.

Figure S4. Relationships between inner bundle sheath cell size and overall bundle sheath areas. Total bundle sheath (BS) area per segment is plotted against the average area of individual BS cells on (A) secondary, (B) tertiary, and (C) minor veins. The total BS area for each vein order is plotted against the average area of individual BS cells on the same vein order for (D) secondary, (E) tertiary, and (F) minor veins. For all plots, C_3 accessions are in blue, C_3-C_4 in green, and C_4 in red. Linear relationships are plotted in black along the whole dataset for all plots, except plot E where the relationship is plotted separately for C_4 (red) and non-C_4 (black) accessions.

Figure S5. Comparison of bundle sheath size in African and non-African C_4 accessions. Mean area of inner bundle sheath cells (i.e., mestome sheath cells) in C_3, C_3-C_4, C_4 African, and C_4 non-African *Alloteropsis semialata* accessions (n=17, 6, 20, and 7, respectively).

Figure S6. Plasticity for leaf anatomical components differs by photosynthetic type. Vein density (*i.e.,* veins per segment), average area of individual bundle sheath (BS) cells on tertiary veins, average number of mesophyll (M) cells between all vein orders, and leaf thickness are plotted for C_3 (blue), C_3-C_4 (green), and C_4 (red) accessions of *Alloteropsis semialata* grown in (A-D) their native field *vs*. controlled environment and (E-H) low *vs*. ambient CO_2 concentrations. Data are means ± 1 SE. T-tests were performed separately by

photosynthetic type to determine whether leaf anatomy differed in the two environments. Where significant, *t* and *p* statistics are provided in the relevant color. Non-significant relationships are denoted with NS.

Figure S7. Organelle abundance differs between photosynthetic types. Mesophyll (A, C, E) and bundle sheath (B, D, F) cell anatomy of C_3 (A-B), C_3-C_4 (C-D), and C_4 (E-F) *A. semialata*. Black and white asterisks label mitochondria and peroxisomes, respectively; +, chloroplast. Scale bars are 2 µm.

Figure S8. Immunodetection of GLDH. Presence of glycine decarboxylase H sub-protein (GLDH) in the mesophyll (A, C, E) and bundle sheath (B, D, F) of C_3 (A-B), C_3-C_4 (C-D), and C_4 (E-F) *Alloteropsis semialata*. Black and white asterisks label mitochondria and peroxisomes, respectively; +, chloroplast. Scale bars are 100 nm. The detection of GLDH follows Khoshravesh *et al.* 2017, except that the blocking time was 30 min in 0.5% BSA in PBS and primary and secondary antibody dilutions were 1:100 (3 h) and 1:20 (1 h), respectively. The Anti-GLDH antiserum was obtained by Agrisera (Vännäs, Sweden) against the recombinant protein from *Flaveria bidentis*.

Figure S9. Immunodetection of Rubisco large subunit. Presence of the Rubisco large subunit in the mesophyll (A, C, E) and mestome sheath cells (B, D, F) of C_3 (A, B), C_3-C_4 (C, D), and C_4 (E, F) *Alloteropsis semialata*. C, chloroplast. Bar, 500 nm. The detection of RBCL follows Khoshravesh *et al.* 2017. The anti-RBCL antiserum was obtained from Agrisera Antibodies (Vännäs, Sweden).

SUPPORTING INFORMATION TABLES

Table S1. Geography, stable carbon isotope ($\delta^{13}C$), CO_2 compensation point (CCP), and phylogenetic clade details used to determine photosynthetic pathway for each *Alloteropsis semialata* accession. $\delta^{13}C$ distinguish C_4 (> -16) from non-C_4 (< -17). C_3-C_4 are distinguished from C_3 via country, CCP, and clade.

Accession	Country	$\delta^{13}C$[a]	CCP	Clade	Pathway
GMT-1	South Africa	-29.10	42.6	Southern Africa C_3[e]	C_3
KWT-3	South Africa	-26.30	44.7[b]	Southern Africa C_3[e]	C_3
ZIM 02-5	Zimbabwe	-26.61	NA	Southern Africa C_3[e]	C_3
ZIM 03-01	Zimbabwe	-25.69	42.2	Southern Africa C_3[e]	C_3
CRL-4-2	South Africa	-24.80	54.8[b]	Southern Africa C_3[c]	C_3
KSD	South Africa	-28.00	NA	Southern Africa C_3[c]	C_3
LSU-3	South Africa	-25.70	NA	Southern Africa C_3[c]	C_3
MDB	South Africa	-23.16	NA	Southern Africa C_3[c]	C_3
MTP 2	South Africa	-26.00	NA	Southern Africa C_3[c]	C_3
SNR	South Africa	-27.76	NA	Southern Africa C_3[c]	C_3
JMS-1	South Africa	-26.80	52.8[b]	Southern Africa C_3[d]	C_3
ASM	South Africa	-26.35	NA	NA	C_3
BST	South Africa	-25.74	NA	NA	C_3
EML-11-3	South Africa	-26.25	49.5[b]	NA	C_3
MR	South Africa	-27.99	NA	NA	C_3
SFB	South Africa	-26.55	NA	NA	C_3
ZIM 04-01	Zimbabwe	-26.10	NA	NA	C_3
L01-A	Tanzania	-26.30	19.4[b]	Central Africa C_3-C_4[e]	C_3-C_4
L04-D	Tanzania	-23.10	25.6[b]	Central Africa C_3-C_4[e]	C_3-C_4
TAN 02-01	Tanzania	-24.62	10.5	Central Africa C_3-C_4[e]	C_3-C_4
TAN 04-16	Tanzania	-25.07	NA	Central Africa C_3-C_4[e]	C_3-C_4
ZAM 03-03	Zambia	-31.06	NA	Central Africa C_3-C_4[e]	C_3-C_4
ZAM 07-02	Zambia	-22.25	9.1	Central Africa C_3-C_4[e]	C_3-C_4
PHL	Philippines	-15.85	1.7	Asian/Australian C_4[e]	C_4
QSLD	Australia	-12.10	5.1[b]	Asian/Australian C_4[e]	C_4
TWN 10	Taiwan	-14.60	4.6	Asian/Australian C_4[e]	C_4
BF	Burkina Faso	-11.20	5.2[b]	African C_4[e]	C_4
MAJ	Madagascar	-11.80	12.3[b]	African C_4[e]	C_4
MDG-1	South Africa	-13.70	5.6[b]	African C_4[e]	C_4
SFD-1-3	South Africa	-12.40	5.2[b]	African C_4[e]	C_4
CAM 1-3	Cameroon	-12.30	2.4	NA	C_4
L02-M	Tanzania	-11.40	NA	African basal C_4[e]	C_4
TAN 03-01	Tanzania	-12.03	2.9	African basal C_4[e]	C_4
AUS 09-01	Australia	-14.38	NA	NA	C_4
AUS 10-01	Australia	-13.94	NA	NA	C_4
AUS 18-01	Australia	-14.41	NA	NA	C_4
AUS 25-01	Australia	-12.82	NA	NA	C_4
GB61	Madagascar	-13.67	NA	NA	C_4
MOZ 01	Mozambique	-13.16	NA	NA	C_4
MSV 1935A	Madagascar	-12.62	NA	NA	C_4
MSV 1937	Madagascar	-12.91	NA	NA	C_4
MSV 2081	Madagascar	-13.02	NA	NA	C_4

Accession	Country	$\delta^{13}C^a$	CCP	Clade	Pathway
ZAM 01-01	Zambia	-12.00	5.4	NA	C_4
ZAM 04-01	Zambia	-12.22	2.9	NA	C_4
ZAM 05-01	Zambia	-13.01	3.4	NA	C_4
ZAM 06-01	Zambia	-12.10	1.5	NA	C_4
ZAM 07-16	Zambia	-13.19	3.5	NA	C_4
ZAM 08-01	Zambia	-12.52	1.7	NA	C_4
ZAM 09-02	Zambia	-13.70	3.0	NA	C_4
ZAM 10-01	Zambia	-12.59	2.4	NA	C_4

[a]The carbon isotope composition of plant tissues ($\delta^{13}C$) was measured on dried leaf tissue using an ANCA GSL preparation module coupled to a 20–20 stable isotope analyser (PDZ Europa, Cheshire, UK). Plants with $\delta^{13}C$ values higher than -17‰ were considered to have a fully functioning C_4 system, while those with values lower than this threshold were considered either C_3 or C_3-C_4. [b]Lundgren *et al.* 2016. Evolutionary implications of C_3-C_4 intermediates in the grass *Alloteropsis semialata. Plant, Cell & Environment* 39: 1974-1885. [c]Lundgren *et al.* 2015. Photosynthetic innovation broadens the niche within a single species. *Ecology Letters* 18: 1021-1029. [d]Olofsson *et al.* 2016. Genome biogeography reveals the intraspecific spread of adaptive mutations for a complex trait. *Molecular Ecology* 25: 6107–6123. [e]unpublished.

Table S2. Details of the *Alloteropsis semialata* accessions used in the plasticity experiment and the environmental conditions at their field collection site. MAT, mean annual temperature; MAP, mean annual precipitation; T, temperature.

**Accession**	**Pathway**	**Country**	**Light**	**MAT (°C)**	**Max T (°C)**	**Min T (°C)**	**Growing Season T (°C)**	**MAP (mm)**
CRL-4-2	C_3	South Africa	Full sun	14.8	26.7	1.3	17.9	828
EML-11-3	C_3	South Africa	Full sun	14.1	26.2	0.2	17.3	743
KWT-3	C_3	South Africa	Full sun	17.0	27.1	7.2	17.7	753
ZIM-2-5	C_3	Zimbabwe	Full sun	14.4	22.9	4.1	17.1	1265
ZIM-3-1	C_3	Zimbabwe	Full sun	15.5	23.9	4.9	18.3	1088
LO1-A	C_3-C_4	Tanzania	Partial shade	23.6	31.7	15.2	23.4	934
LO4-A	C_3-C_4	Tanzania	Partial shade	16.6	24.9	7.2	17.7	1415
TAN-2-1	C_3-C_4	Tanzania	Partial shade	16.7	25.0	7.1	17.1	957
ZAM-7-2	C_3-C_4	Zambia	Partial shade	18.9	29.0	6.2	19.6	1365
CAM-1-3	C_4	Cameroon	Full sun	22.7	29.0	15.2	21.6	1934
MDG-1	C_4	South Africa	Full sun	17.3	29.8	2.8	20.6	675
SFD-4	C_4	South Africa	Full sun	14.2	26.0	1.3	18.6	759
TAN-3-1	C_4	Tanzania	Partial shade	20.2	28.5	11.0	20.6	972

Accession	Pathway	Country	Light	MAT (°C)	Max T (°C)	Min T (°C)	Growing Season T (°C)	MAP (mm)
ZAM-1-1	C_4	Zambia	Partial shade	20.1	29.0	10.2	20.3	1205
ZAM-10-1	C_4	Zambia	Partial shade	19.9	30.4	6.0	21.2	1103
ZAM-5-1	C_4	Zambia	Full sun	19.9	29.6	7.4	20.5	1224
ZAM-9-2	C_4	Zambia	Partial shade	19.1	30.4	4.6	20.2	1211
			Mean C_3	15.2	25.4	3.5	17.7	935
			Mean C_3-C_4	18.9	27.7	8.9	19.4	1168
			Mean C_4	19.2	29.1	7.3	20.5	1135
			Minimum	14.1	22.9	0.2	17.1	675
			Maximum	23.6	31.7	15.2	23.4	1934
